# *Ogeramua*, a new name for the land snail genus *Papuanella* Clench & Turner, 1959 (Mollusca, Gastropoda, Camaenidae), preoccupied by *Papuanella* Distant, 1914 (Insecta)

**DOI:** 10.3897/zookeys.678.13468

**Published:** 2017-06-07

**Authors:** Carl C. Christensen

**Affiliations:** 1 Bernice P. Bishop Museum, 1525 Bernice Street, Honolulu, Hawaii 96821, USA

**Keywords:** Camaenidae, zoological nomenclature, homonymy, Papua New Guinea, *Papuanella*, *Ogeramua*

## Results

The genus *Papuanella* (type species *Geotrochus ogeramuensis* Kobelt, 1914, by original designation) was established by Clench and Turner (1959: 5) for two species of land snails inhabiting the Central Highlands of Papua New Guinea. *Papuanella* is currently recognized as a valid genus within the subfamily Papuininae of the family Camaenidae (Schileyko 2003).

Distant (1914: 352) established *Papuanella* for a genus of fulgorid homopterans also inhabiting the island of New Guinea, and thus Clench and Turner’s taxon is a junior homonym in need of replacement. Accordingly, pursuant to Article 60 of the International Code of Zoological Nomenclature (International Commission on Zoological Nomenclature 1999), the name *Ogeramua*, nom. n., is here proposed as a replacement name for *Papuanella* Clench & Turner, 1959 (non *Papuanella* Distant, 1914). Species included in the genus as defined by Clench and Turner (1959) are *O. ogeramuensis* (Kobelt, 1914), comb. n., and *O. finisterrensis* (Kobelt, 1914), comb. n.

The epithet *Ogeramua* is named for Ogeramua, Papua New Guinea, the type locality of its type species, and is to be treated as feminine in gender.
